# Supplementary material for: Prevalence of anxiety and depression symptoms and associated factors among women diagnosed with breast cancer: A cross-sectional study at Ocean Road Cancer Institute in Dar es Salaam–Tanzania
Source: PLoS One. 2025 Jul 3;20(7):e0326749. doi: 10.1371/journal.pone.0326749 (PMC12225821; doi:10.1371/journal.pone.0326749)
Supplement: Attachment — Submitted filename: Ummy Msengas Response to Reviewers.docx [file pone.0326749.s002.docx]

**PONE-D-24-37916**

Prevalence of anxiety and depression symptoms and associated factors among women diagnosed with breast cancer: A cross-sectional study at Ocean Road Cancer Institute in Dar-es-Salaam - Tanzania.

PLOS ONE

**Corresponding author’s name:** Ummy Msenga

I deeply appreciate the review and taking the necessary time and effort to notice areas of improvement in my manuscript document. I sincerely appreciate the valuable comments and suggestions, they have surely enriched me with more knowledge on how to improve the quality of my manuscript.

| Comments | Explanation | Page No: |
| --- | --- | --- |
| The introduction adequately sets the stage by presenting the significance of anxiety and depression in breast cancer patients, particularly in low-income countries. However, it could strengthen the rationale by expanding on the gap in local Tanzanian research beyond referencing socioeconomic factors. A clearer link between this gap and the current study would add value. | Thank you for your comment. However, there is limited data from local Tanzanian research. Maghina et al, 2022 is the only article that addressed psychological factors associated with anxiety and depression symptoms among women diagnosed with breast cancer receiving chemotherapy. The results from Maghina’s study highlighted socioeconomic factors. | 4 |
| It would be beneficial to compare the prevalence rates found in this study with global or regional studies more thoroughly in the results section. Although global prevalence is briefly mentioned in the introduction, it would provide a more direct assessment of whether the rates observed in Tanzania are higher or lower than expected. | Thank you for the comment. The prevalence rates found in this study have been compared by those of global and regional studies in the discussion section. | 20-21 |
| The conclusion appropriately emphasizes the need for mental health screening and intervention, but it could benefit from more specific recommendations on integrating these services into existing cancer care frameworks in Tanzania. | Thank you for the comment. We have incorporated a recommendation on integrating mental health services into the existing cancer care. | 24-25 |
| **Reviewer #1:**  Dear Editor, thank you very much for inviting me to review this important topic that aligns with my specialty. I also appreciate authors for bringing such an interesting and current global concern topic. Even though the manuscript has merit, it needs a major revision, especially on the method and result sections. | Thank you very much for your observations. |  |
| 1. In the abstract section, please coordinate those mental health problems with breast cancer to attract the reader... | Thank you for your observation. The abstract section has been rewritten to coordinate anxiety and or depression with breast cancer. | 2 |
| 2. Your data collection period is between May and 17 June 2023; please specify the date. | We appreciate your observation, the date has been specified. | 6 |
| 3. Your recommendation is poor. Please rewrite your recommendation based on your findings. | Thank you for the comment. Our conclusion and recommendation has been rewritten to align with our findings. | 24-25 |
| 4. In sample size calculation, why you didn’t add non-response rate... | Thank you for your input. We did not include the non-response rate during sample size calculation because we used interviewer administered questionnaire method to collect data. This method offers an increased engagement between researcher and participant, opportunity to give clarification and motivation for participants to answer questions, ability to follow up on unanswered question and therefore lowering the non-response rate significantly. | 5 |
| 5. Please clearly put your operational definitions... | We appreciate your suggestion. Our operational definitions are clearly put in the introduction section. | 3 |
| 6. How do you select your study participants? Who diagnosed your study participants??? | Thank you for your question. The researcher and or research assistant used patients registry book to identify all patients who were eligible for the study.  Researcher diagnosed study participants with anxiety and or depression symptoms using Hospital Anxiety and Depression Scale (HADS) immediately after completion of filling out the questionnaire. | 6 & 9-10 |
| 7. In the result section, your response rate was 100%... Are you sure??? | Thank you.  The researcher used interviewer administered questionnaire during data collection which is considered the gold standard survey mode. In addition research assistants were well trained to make sure that they collect quality data. |  |
| 8. It’s not clear how you categorize the variables age, marital status, and educational status. | Thank you for your observation. We categorized the variable age using WHO 2015 age criteria.  We categorized the marital status according to Fatiregun, et al 2016.  The Tanzania's education system is based on the British system and includes pre-primary, primary, secondary, and vocational education. Hence we used classifications similar as those from Maghina, et al 2022. | 11 |
| 9. Who reports the stage of cancer???? | Thank you for your question. We wanted to identify if knowing or not knowing one’s stage of cancer is associated with anxiety and or depression. Therefore, the patients were asked to mention their stages of cancer. |  |
| 10. In binary tables, how do you select the variables for multivariable? For instance, age is not significantly associated with the crude odds ratio, so how is it a candidate for AOR??? | Thank you for your observation. It is true that age is not significantly associated with the crude odds ratio yet, according to literature it is a confounder hence making it candidate for the multivariable analysis. |  |
| 11. Please revise BINARY TABLE... | Thank you for your observation. We have revised the binary logistic regression analysis and results table. | 17-19 |
| 12. Discussion is poor. | Thank you for your observation. The discussion has been rewritten to be more focused. | 20-23 |
| 13. Rewrite your conclusion and recommendations... It needs revision. Your recommendation should align with your findings. | Thank you for the comment. Our conclusion and recommendation is rewritten to align with the findings of our study. | 24-25 |
| **Reviewer #2:**  I do not have major concern with the technical content of the paper. However, there are some concerns a) The introductioon section did not provide convincing justification on the need for this study. Given there are already available evidences on prevamence of anxiety and depression in conneciton to breast cancer, why should this study be of interest? This is not clear from the introduction | Thank you for you observation. It is true that there are already available evidences on prevalence of anxiety and depression in conneciton to breast cancer. However, due to scarcity of data from Tanzania, this paper adds data to the existing literature. | 4 |
| 2) The findings from this study presented factors associated with and anxiety and depression. However, similar findings are out there in Sub-Saharan Countries. Again these findings are not any different from what is availble. Again this is critical limitaiton of this study | Thank you for the observation. It is true that there are similar findings out there in the Sub-Saharan countries. However none exists in Tanzania, hence as this study represents women diagnosed with breast cancer in Tanzania, it adds data to the existing literature. | 4 |
| 3) the conclusive argument on implicaion of anxiety and depression doesn't seem to offer any useful advice to researchers, policy makers and programmers. So, this study doesn't have novelity. | We appreciate your observation. We have rewritten the conclusion. | 24-25 |
| **Reviewer #3:**  GENERAL COMMENTS/ QUERIES/ RECOMMENDATIONS:  1- A good and important topic for clinical and public health domains. | Thank you very much for your observation. |  |
| 2- A vote of thanks to authors for considering "patients' perspectives" in their clinical research findings. A commendable attitude! | Thank you very much for your observation. |  |
| 3. However, there are notable errors/questionable statements/sentences that are at best contradictory and at worst fallacious in composition throughout the manuscript sections! e.g. in results section, what has been reported as “bivariate analyses” are in actual fact UNIVARIATE/CRUDE ANALYSES and what went on as “multivariate analyses” in actual fact were MULTIVARIABLE ANALYSES!  Besides, findings of univariate & multivariable binary logistic regression suggest POTENTIAL BUT SIGNIFICANT confounding!!! | Thank you for your comment. As per our understanding bivariate analysis is the one that involves the analysis of two variables (often denoted as x,y) for the purpose of determining the empirical relationship between them.  Univariate analysis is where only one variable is examined at a time allowing the researcher to describe and summarize the characteristics of that single variable within a data set without considering relationship between multiple variables.  Crude analysis is a basic or initial examination of raw data without any significant manipulation or refinement, providing a rough over view of the information without considering complex factors or adjustments.  According to the above explanation we believe we did bivariate analysis since, for example we took each dependent variable such as anxiety symptoms against each independent variable such as age at a time.  However we agree with the reviewer that what was supposed to be done and what we did was multivariable analysis rather than multivariate analysis. Hence we have replaced multivariate analysis with multivariable analysis in this version. | 15-19 |
| Otherwise, it was not clear (and some justifications FALLACIOUS!) on how/which criteria were used to dichotomise variables during analysis. For instance, it is not true that UN considers elderly people as >61 years old (fact: people >65 years are considered 'elderly' under current UN definition) | Thank you for your observation. It is true that the current UN categories of age are not similar to the one we used because by then we used age categories according to WHO 2015, which classified elderly people as those above 60 years old. | 10 |
| Recommendations: MINOR correction but consider (in addition to specific comments attached separately to editors):  1. to re-write the manuscript (see specific comments/queries/recommendations attached to editors!) once again before it could MERIT consideration for publication at PLOS ONE journal. | Thank you very much. We have re-written the manuscript following comments, queries and recommendations provided. |  |
| 2. Authors should attach RAW DATASET (preferably as an APPENDIX) for verification of their analyses in lieu of the current picture displayed in the results section of the manuscript! | We appreciate your suggestion. The raw data set is attached with the resubmission of my manuscript as an appendix. |  |
